# Cooperation between RUNX1-ETO9a and Novel Transcriptional Partner KLF6 in Upregulation of *Alox5* in Acute Myeloid Leukemia

**DOI:** 10.1371/journal.pgen.1003765

**Published:** 2013-10-10

**Authors:** Russell C. DeKelver, Benjamin Lewin, Kentson Lam, Yukiko Komeno, Ming Yan, Chandler Rundle, Miao-Chia Lo, Dong-Er Zhang

**Affiliations:** 1 Division of Biological Sciences, University of California San Diego, La Jolla, California, United States of America; 2 Department of Biomedical Sciences, University of California San Diego, La Jolla, California, United States of America; 3 Moores Cancer Center, University of California San Diego, La Jolla, California, United States of America; 4 Department of Pathology, University of California San Diego, La Jolla, California, United States of America; Cincinnati Children's Hospital Medical Center, United States of America

## Abstract

Fusion protein RUNX1-ETO (AML1-ETO, RUNX1-RUNX1T1) is expressed as the result of the 8q22;21q22 translocation [t(8;21)], which is one of the most common chromosomal abnormalities found in acute myeloid leukemia. RUNX1-ETO is thought to promote leukemia development through the aberrant regulation of RUNX1 (AML1) target genes. Repression of these genes occurs via the recruitment of the corepressors N-COR and SMRT due to their interaction with ETO. Mechanisms of RUNX1-ETO target gene upregulation remain less well understood. Here we show that RUNX1-ETO9a, the leukemogenic alternatively spliced transcript expressed from t(8;21), upregulates target gene *Alox5*, which is a gene critically required for the promotion of chronic myeloid leukemia development by BCR-ABL. Loss of *Alox5* expression reduces activity of RUNX1-ETO9a, MLL-AF9 and PML-RARα *in vitro*. However, *Alox5* is not essential for the induction of leukemia by RUNX1-ETO9a *in vivo*. Finally, we demonstrate that the upregulation of *Alox5* by RUNX1-ETO9a occurs via the C_2_H_2_ zinc finger transcription factor KLF6, a protein required for early hematopoiesis and yolk sac development. Furthermore, *KLF6* is specifically upregulated by RUNX1-ETO in human leukemia cells. This identifies KLF6 as a novel mediator of t(8;21) target gene regulation, providing a new mechanism for RUNX1-ETO transcriptional control.

## Author Summary

The 8;21 translocation is one of the most common genetic abnormalities present in acute myeloid leukemia (AML). This translocation causes expression of the fusion gene *RUNX1-ETO* and its splicing isoforms. RUNX1-ETO proteins then reprogram the transcriptional landscape of the cell and cooperate with further mutations to induce leukemia development. In this study, we examine the transcriptional control of the RUNX1-ETO target gene *Alox5*. Although *Alox5* appears to be dispensable for AML development in a mouse model, it is required for some RUNX1-ETO functions. In studying the regulation of Alox5 expression, we have discovered a novel RUNX1-ETO partner protein, KLF6, which is both upregulated by RUNX1-ETO and participates in RUNX1-ETO gene regulation. This provides new insight into the under-studied mechanisms of RUNX1-ETO target gene upregulation and identifies KLF6 as a potentially important protein for further study in t(8;21) AML development.

## Introduction

Acute myeloid leukemia (AML) is the most prevalent form of adult leukemia [1]. Chromosomal translocations are found in over 80% of AML, the most common of which is t(8;21), occurring in up to 40% of AML cases categorized within the French-American-British (FAB) subtype M2 [2]–[6]. This translocation results in the expression of fusion protein RUNX1-ETO. Although sufficient for *in vitro* immortalization, RUNX1-ETO requires additional cooperating mutations to induce leukemia *in vivo* [7]–[9]. RUNX1-ETO also exists as C-terminally truncated forms due to alternative splicing at exons 9 (RUNX1-ETO9a) and 11 (RUNX1-ETO11a) [10], [11]. Both isoforms lack the NHR4/MYND domain and are expressed in human t(8;21)+ leukemia patient samples, and RUNX1-ETO9a (RE9a) strongly promotes leukemia development in mice [10], [11].

RUNX1-ETO (RE) is known to be a transcriptional repressor through its recruitment of the corepressors N-CoR and SMRT and their associated histone deacetylases [12]–[14]. RE can also activate promoters cell-specifically, however it is unclear whether such gene activation occurs in a direct or indirect fashion [15]. The mechanisms by which RE upregulates its target genes have been less thoroughly investigated, although recent studies have utilized ChIP-chip and ChIP-seq to identify putative RE target genes to examine their regulation and importance in leukemia development [16]–[18]. One interesting recent finding is that RE upregulates at least some of its target genes via its interaction with the histone acetyltransferase p300, and that loss of this interaction significantly delays leukemia onset [19]. Additionally, our group recently reported that RE9a recruits PRMT1 to some RE9a-activated genes, leading to H3K4 methlyation, H3K9/14 acetylation and transcriptional activation [20]. Further mechanisms of gene upregulation remain to be investigated.

One gene strongly upregulated in t(8;21) leukemia is *ALOX5*, encoding an enzyme required for the synthesis of leukotrienes, which are small, lipid-derived signaling molecules that trigger pathways implicated in both inflammation and cancer, such as proliferation, cell survival and angiogenesis [21]–[24]. In fact, inhibitors of the ALOX5 pathway have shown promise in treating a number of epithelial cancers [25]–[27]. In addition, ALOX5 has previously been shown to function in both normal hematopoiesis and leukemia development. Using a human CD34^+^ cell model, it has been demonstrated that leukotrienes both increase proliferation and exert an anti-apoptotic effect on human hematopoietic stem cells [28]. More recently, it was further demonstrated that ALOX5 is required for the induction of chronic myeloid leukemia (CML) by BCR-ABL, and a specific ALOX5 inhibitor is able to significantly delay CML onset when used either alone or in combination with the BCR-ABL kinase inhibitor imatinib [29].

Despite significant advances in our molecular understanding of AML, frontline treatment for this disease is still induction and consolidation chemotherapy, similar to the protocol established 30 years ago, and overall survival for older patients has not improved over the same time period [30], [31]. Furthermore, although t(8;21)+ AML is considered to have a favorable prognosis for chemotherapeutic response, the 10-year overall survival for patients with this cytogenetic signature is only 61% [32]. New molecular targets and a better understanding of the mechanisms of RUNX1-ETO-mediated transcriptional changes leading to disease development are needed for safer and more effective treatment of this disease. Here we investigate the role of ALOX5 in AML development, establishing *ALOX5* as an upregulated gene in t(8;21) leukemia that is also important in cellular dysregulation by multiple oncogenic fusion proteins. We further discover that RE9a upregulates *Alox5* via the C_2_H_2_ zinc finger transcription factor Krϋppel-like factor 6 (KLF6), a protein critically required for early hematopoiesis [33]. Finally, KLF6 itself is upregulated by both RE and RE9a, establishing a new mechanism for the upregulation of target genes by t(8;21) fusion proteins and a new pathway to study in AML development.

## Results

### Upregulation of *ALOX5* in t(8;21)-associated acute myeloid leukemia

**Figure 1:**
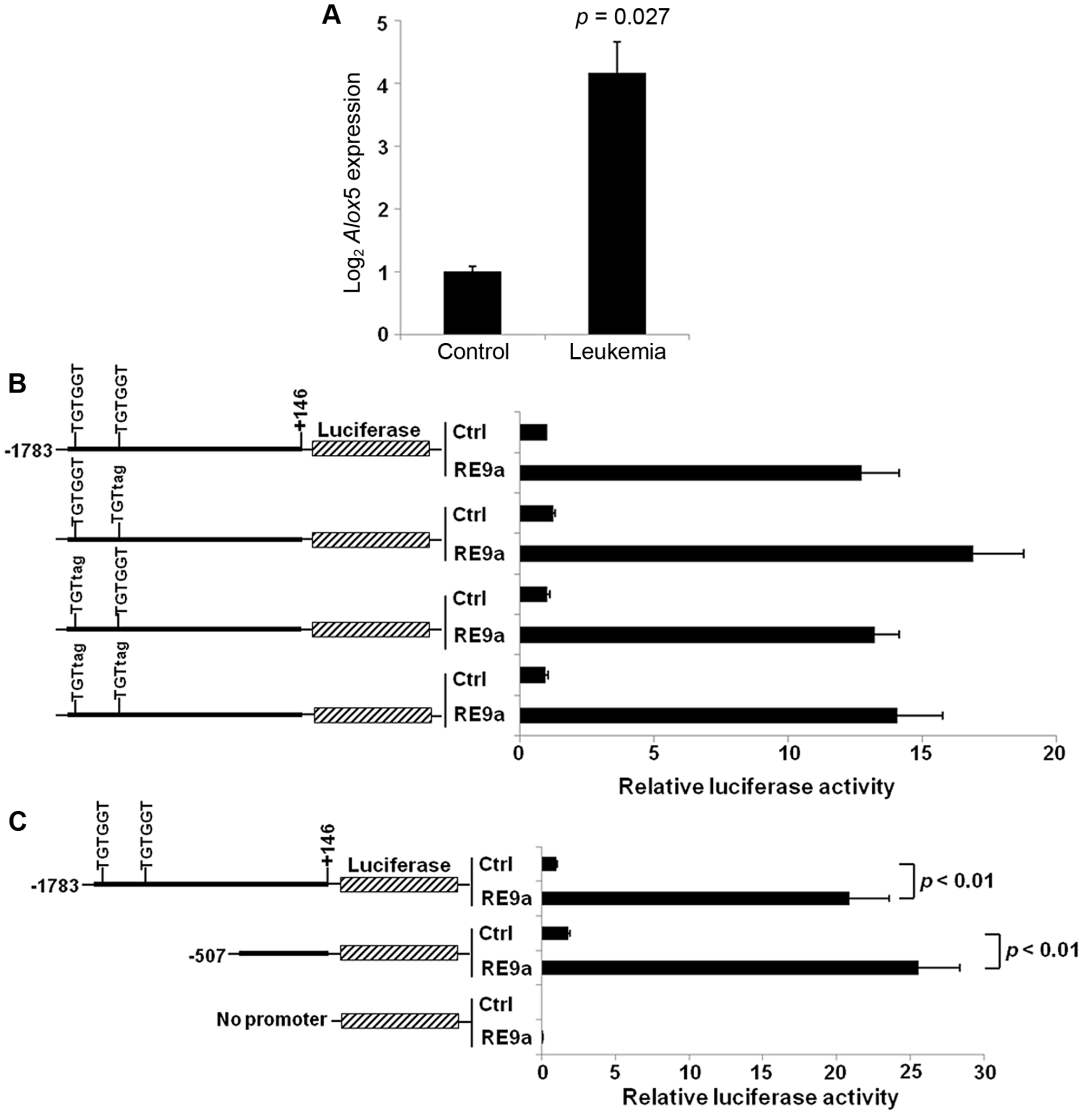
Upregulation of *Alox5* in acute myeloid leukemia and by RUNX1-ETO9a. (A) Normalized log_2_ expression of *Alox5* in control or RE9a-leukemic murine lin^−^c-Kit^+^ bone marrow cells. mRNA transcript levels were normalized to *Gapdh* and control was set to 1. Data show averages and standard deviations from 3 independent mice each. (B) RE9a regulation of mouse *Alox5* promoter-luciferase reporter. Numbers indicate base pair relative to transcription start site. Two RUNX1 binding sites (TGTGGT) were either wildtype or mutated to TGTtag to abrogate RE9a binding [54]. Indicated promoter-luciferase reporter was co-transfected with control (Ctrl) or RE9a plasmid and expression was normalized to *Renilla* luciferase. Wildtype promoter+control set to 1. (C) RE9a regulation of truncated mouse *Alox5* promoter-luciferase reporter. Luciferase assay performed as described in (B), with −1783 to +146 promoter+control set to 1.

In order to understand the mechanism by which RUNX1-ETO (RE) contributes to t(8;21) acute myeloid leukemia (AML) development, our group recently conducted gene expression microarray and ChIP-chip analyses to identify potential disease-related RE target genes [18]. One gene confirmed to be highly upregulated and specifically detected by ChIP in the RE9a murine leukemia model is *Alox5*. The detected ChIP peak is 47 kilobases downstream of the transcription start site in intron 9. ALOX5 is required for CML development due to the depletion of leukemia stem cells in the absence of *Alox5* expression [29]. To determine whether *ALOX5* is also upregulated in human t(8;21) AML, we analyzed publicly available microarray data of AML M2 patients with or without the 8;21 translocation [34]. As shown in Supporting Figure S1A, *ALOX5* expression is approximately 2.3 fold higher in t(8;21)+ patients than in patients not harboring the translocation, and both patient groups had elevated *ALOX5* levels relative to normal CD34+ control samples. This upregulation correlates very well to the *Alox5* upregulation seen in the RE9a mouse model (Figure 1A).

As an initial step in determining the molecular mechanism by which *Alox5* is upregulated, we cloned a ∼1.9 kb *Alox5* promoter fragment (bp −1783 to +146) upstream of a luciferase reporter. The vector backbone of this reporter contained 6 RUNX1 consensus binding sites which were mutated to prevent non-specific regulation by RE9a due to binding at these sites [15]. In promoter-luciferase transactivation studies, the *Alox5* promoter is strongly upregulated by RE9a (Figure 1B). This promoter fragment contains two consensus RUNX1 binding sites (TGTGGT), indicating that RE9a may bind directly to the promoter at these sites to affect gene expression. However, when these sites are mutated either individually or together, RE9a is still able to upregulate the reporter (Figure 1B). Furthermore, when the promoter region is truncated to exclude these two RUNX1 binding sites, there is no decrease in the activation of the *Alox5* promoter by RE9a (Figure 1C), implying that RE9a either does not upregulate *Alox5* directly or functions via binding at a non-canonical RUNX1 binding site.

### RE9a cooperates with KLF6 to upregulate *Alox5*

**Figure 2:**
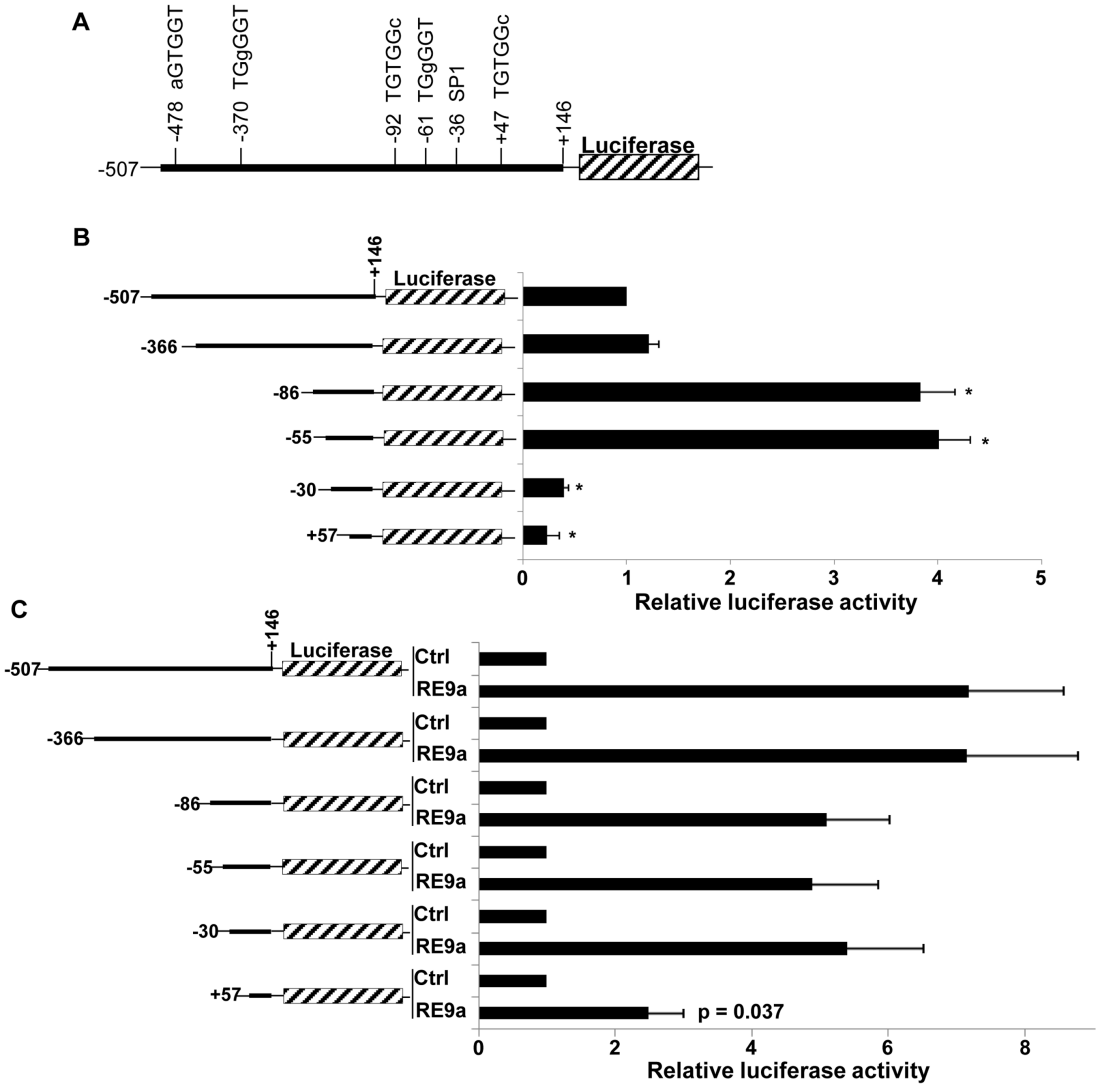
Analysis of *Alox5* promoter regulation by RE9a. (A) Schematic of *Alox5* promoter-luciferase reporter with motifs differing from RUNX1 consensus binding site (TGYGGT) indicated. Bases differing from consensus site labeled in lowercase. Transcription factor SP1 binding site also indicated. Numbers represent base pairs relative to transcription start site. (B) Basal regulation of *Alox5* promoter-luciferase truncations. Indicated reporters were transfected in the absence of RE9a and expression was normalized to *Renilla* luciferase and the −507 to +146 construct was set to 1. * = *p*<0.01 relative to −507 reporter. (C) Inducible regulation of *Alox5* promoter-luciferase by RE9a. Indicated reporters were co-transfected with control or RE9a and expression was normalized to *Renilla* luciferase. Each control (Ctrl) transfection normalized to 1. *p*-value relative to −507 reporter +RE9a.

To determine if RE9a upregulates *Alox5* through an imperfect RUNX1 binding site, we examined the truncated promoter from Figure 1C and found 5 motifs that differ from the consensus RUNX1 binding site (TGYGGT) by a single nucleotide (Figure 2A). We then serially truncated this promoter fragment to examine which regions are important for both the basal and RE9a-inducible expression of *Alox5*. When truncated from −366 to −86, we find a significant increase in the basal activity, indicating the presence of a basal *cis* repressive element in this region (Figure 2B). When truncated from −55 to −30, there is a large reduction in basal activity, which is unsurprising as this truncation removes an SP1 binding site (GGGCGG) known to be important for *Alox5* expression [35]. When examining inducible activity, however, only the shortest truncation shows a significant loss of promoter activation upon addition of RE9a (Figure 2C), implying that sequences between −30 and +57, which is the region typically considered to comprise the core promoter for gene expression [36], play an important role in *Alox5* regulation by RE9a.

**Figure 3:**
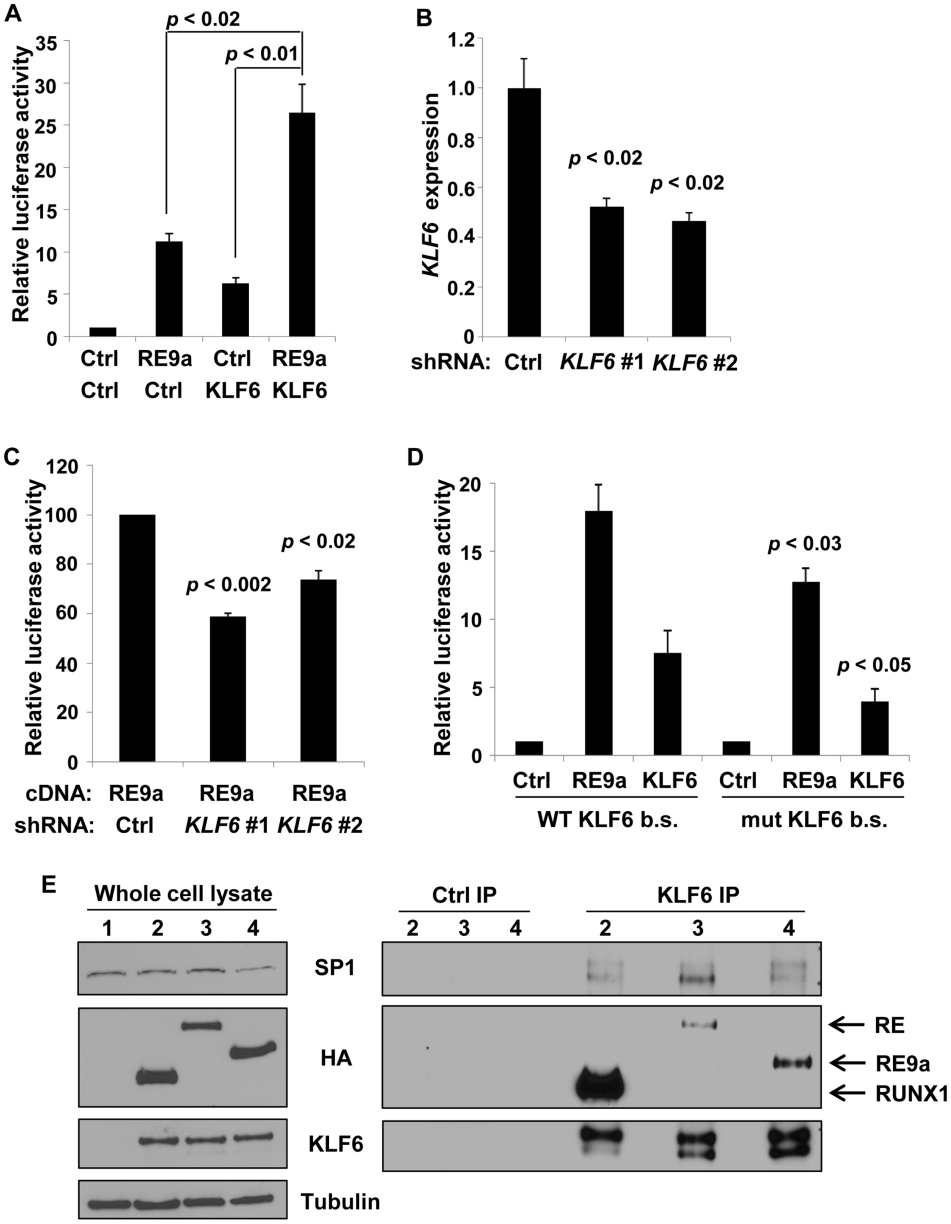
*Alox5* regulation by RE9a and KLF6. (A) Coregulation of *Alox5* reporter by RE9a and KLF6. *Alox5* −507 to +146 reporter co-transfected with RE9a, KLF6 or both. Expression was normalized to *Renilla* luciferase and control (Ctrl) was set to 1. (B) Knockdown of endogenous *KLF6* via shRNA. K562 cells were transfected with control or one of two independent shRNAs targeting *KLF6* and analyzed by qRT-PCR for *KLF6* expression. Expression values were normalized to *GAPDH* and control transfected value was set to 1. Data show averages with standard deviations of 3 independent transfections. (C) Knockdown of *KLF6* impairs ability of RE9a to upregulate *Alox5* promoter-luciferase reporter. K562 cells pre-transfected with control or *KLF6* shRNA were co-transfected with *Alox5* −507 to +146 reporter and RE9a. Expression was normalized to *Renilla* luciferase and control +RE9a was set to 100. (D) Mutation of KLF6 binding site significantly decreases activation of *Alox5* promoter by RE9a and KLF6. Wildtype or KLF6 binding site (b.s.)-mutated (GGGTG to GATCG) *Alox5* −30 to +146 reporter co-transfected with RE9a or KLF6. Expression was normalized to *Renilla* luciferase. Control (Ctrl) was set to 1. *p*-values are compared to wildtype reporter co-transfected with corresponding transgene. (E) KLF6 can interact with RUNX1, RE and RE9a. KLF6 and RUNX1, RE or RE9a were co-transfected into K562 cells, and lysates were immunoprecipitated with control or KLF6 antibody. Also shown is interaction with endogenous SP1. α-tubulin serves as a loading control for the whole cell lysate. After IP, KLF6 appears as multiple bands likely because KLF6 is expressed endogenously as multiple splicing isoforms which are enriched to more easily detectable levels by immunoprecipitation [55].

Upon closer examination of this region, we identified a GGGTG motif (reverse complement: CACCC) which is known to be a binding site for KLF6, a zinc finger DNA-binding transcription factor that was previously identified as an important cell-specific positive regulator of Leukotriene C_4_ Synthase (LTC_4_S) expression, which functions downstream of ALOX5 in the synthesis of certain leukotrienes [37]. Although the CACCC motif is a binding site for most KLF family members [38], since KLF6 is both an important hematopoietic regulator and induces expression of another member of the ALOX5 pathway [33], [37], we decided to focus on KLF6 and hypothesized that RE9a may function through KLF6 to regulate *Alox5* expression. Interestingly, we found that KLF6 is both independently capable of activating the *Alox5* reporter and, when introduced in combination with RE9a, the activation is greater than the sum of the effect of RE9a and KLF6 alone (Figure 3A). This demonstrates that RE9a can indeed cooperate with KLF6 to upregulate *Alox5* expression. To examine whether KLF6 is required for RE9a regulation of *Alox5*, we utilized two different shRNAs targeting *KLF6* that decrease its endogenous expression by approximately 50% (Figure 3B). Both of these shRNAs are able to significantly reduce the ability of RE9a to activate the *Alox5* promoter, demonstrating that RE9a does require KLF6 for full regulation of *Alox5* (Figure 3C). Possible explanations for an incomplete loss of reporter induction after *KLF6* knockdown include an incomplete loss of *KLF6* expression (Figure 3B) and that RE9a may function with other factors in addition to KLF6 in the upregulation of *Alox5*. Furthermore, when the KLF6 binding motif was mutated to a sequence previously shown to disrupt KLF6-mediated gene activation [37], the upregulation of *Alox5* by both RE9a and KLF6 was significantly reduced (Figure 3D). Interestingly, although the specific KLF6 binding site GGGTG found in the murine promoter is not conserved in humans, a GC-box (GGGCGGG) is present at this site, which also allows KLF6 binding [39]. Additionally, the human *ALOX5* promoter does contain 7 CACCC or GGGTG motifs within 500 bp of the transcription start site, indicating that KLF6 function in human *ALOX5* regulation may be conserved through these sites. Supporting this possibility, both exogenous Flag-KLF6 and HA-RE9a display enrichment at the *ALOX5* promoter region in K562 cells when examined by ChIP, although endogenous RUNX1 does not (Figure S2). Finally, since RE9a functions with KLF6 in the regulation of gene expression, we were interested to determine whether RE9a and other RUNX1 proteins were capable of interacting with KLF6 in the same complex. Using coimmunoprecipitation, we find that KLF6 is indeed capable of interacting with both RUNX1 and the fusion proteins RUNX1-ETO and RE9a, which is the first report of such interaction (Figure 3E). However, in all conditions tested we can only detect over-expressed, exogenous KLF6 with available antibodies and therefore could not confirm the interactions between KLF6 and these proteins at the endogenous level. We also confirm the previously reported interaction between KLF6 and endogenous SP1 [39]. Given that RUNX1-ETO is also able to interact with SP1 via the Runt domain [40] (which is common to wildtype RUNX1), this raises the possibility that KLF6, SP1 and Runt domain-containing proteins may function together in gene regulation and provides direction for future transcriptional studies.

### *KLF6* is specifically upregulated in t(8;21)+ cells

**Figure 4:**
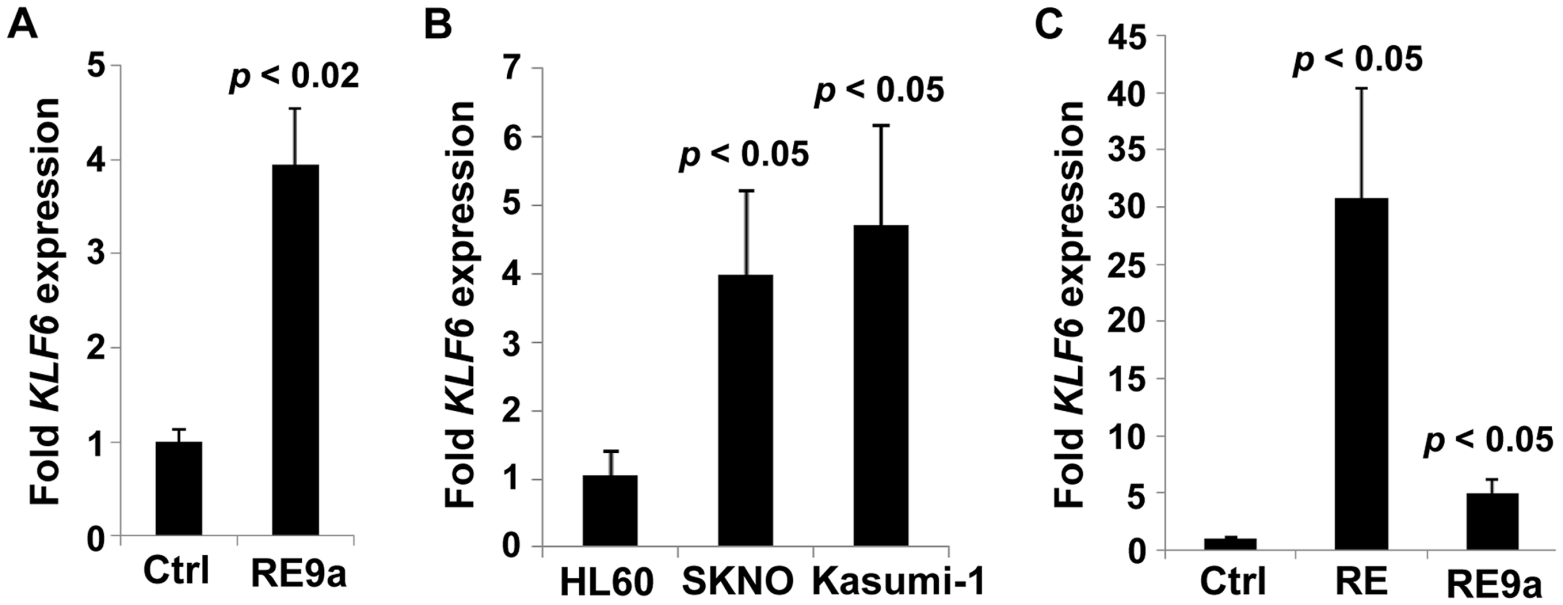
Regulation of *KLF6* by RUNX1-ETO and RE9a. (A) Expression of *KLF6* in control- (Ctrl) or RE9a-transfected K562 cells. RE9a or empty vector was co-transfected into K562 cells along with a GFP-expressing vector to determine transfection efficiency. *KLF6* mRNA levels were normalized to *GAPDH* with Ctrl set to 1, and samples were then normalized to account for transfection rate by percent GFP-expressing cells as determined by flow cytometry. Data show averages and standard deviations of three independent transfections. (B) Expression of *KLF6* in HL60 [t(8;21)-negative] and SKNO and Kasumi-1 [t(8;21)-positive] cell lines. *KLF6* mRNA levels were normalized to *GAPDH* and HL60 was set to 1. Data show averages and standard deviations of 3 independent RNA isolations. (C) Expression of *KLF6* in control-, RUNX1-ETO- or RUNX1-ETO9a-transduced HL60 cells. Following 2 rounds of retroviral transduction, *KLF6* levels determined as in (B), with control-transduced cells (Ctrl) set to 1. Data display averages and standard deviations of 3 independent transductions.

To determine whether *KLF6* is also a target of RE gene regulation in human AML, we again examined available patient data and find that *KLF6* expression is specifically higher in t(8;21)+ leukemia samples (Figure S1B and [34]). *KLF6* is also upregulated in K562 cells 24 hr after transfection with RE9a (Figure 4A), supporting induction of *KLF6* as a mechanism of *Alox5* upregulation by RE9a in the above luciferase studies. Furthermore, when comparing mRNA levels in AML M2 human leukemia cell lines, the t(8;21)+ cell lines SKNO and Kasumi-1 express significantly higher levels of *KLF6* than does the t(8;21)- cell line HL60 (Figure 4B). Finally, when RE or RE9a are introduced retrovirally into HL60 cells, there is a significant and dramatic upregulation of *KLF6* (Figure 4C). This upregulation is greater for RE than RE9a, which agrees with previous findings that RE more strongly dysregulates gene expression than its leukemic isoforms [41], [42]. Collectively, these data demonstrate that *KLF6* is a target of t(8;21) gene upregulation.

### Lack of *Alox5* impairs cellular dysregulation by multiple oncogenic fusion proteins

**Figure 5:**
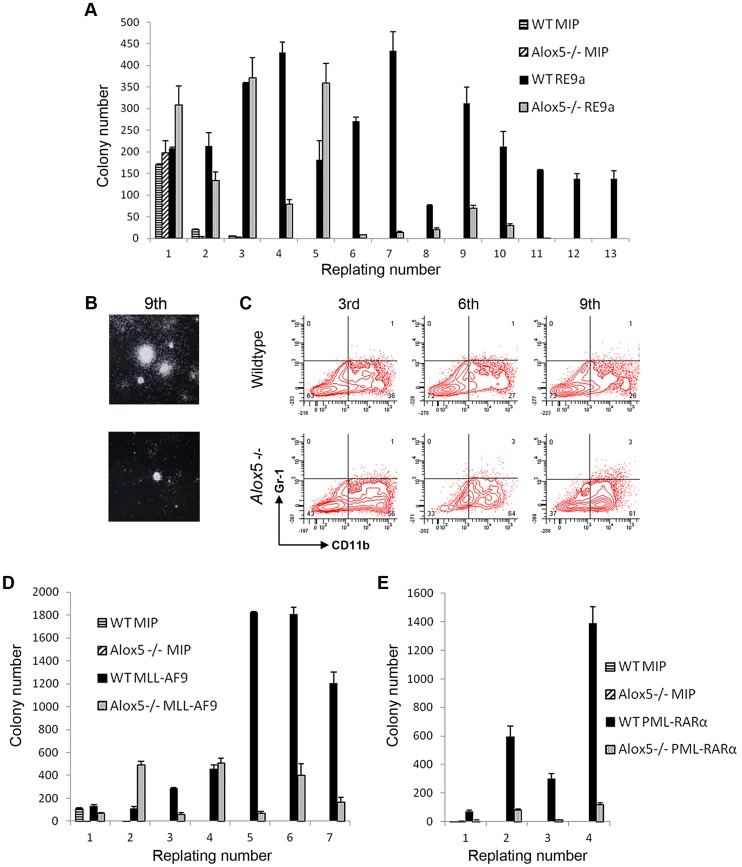
*Alox5* involvement in hematopoietic cell self-renewal. (A) *Alox5* required for long-term self-renewal of hematopoietic cells by RE9a. Colony numbers from wildtype or *Alox5*-/- bone marrow cells transduced with control (MIP) or RE9a retrovirus and serially replated in methylcellulose. Data shown are averages with standard deviations of a representative dataset. Four independent assays were performed. (B) Typical colony images after 9^th^ replating from (A) taken using Nikon Eclipse TS100 microscope with 2×/0.06 objective lens and Nikon DS Camera Control Unit DS-U2 system. (C) Flow cytometric analysis of replated cells from (A). Cells from 3^rd^, 6^th^ and 9^th^ replatings were stained for myeloid lineage markers Gr-1 and CD11b. Representative data from four independent assays shown. (D) and (E) Lack of *Alox5* decreases colony formation potential of hematopoietic cells transduced with MLL-AF9 and PML-RARα. Wildtype or *Alox5*-/- bone marrow cells were transduced with MIP and MLL-AF9 (D) or PML-RARα (E) retrovirus and serially replated in methylcellusose. Data shown are averages and standard deviations of representative datasets. Three independent assays were performed.

To examine the potential functional implications of *Alox5* in t(8;21)-induced self-renewal, we performed serial replating assays after retroviral transduction of wildtype and *Alox5*-/- murine bone marrow cells. In this assay, wildtype cells transduced with retrovirus encoding RE9a-IRES-Puro^r^ display increased self-renewal and maintain replating capacity through at least 13 weeks in weekly replating culture, whereas control cells transduced with a vector encoding the puromycin resistance gene alone (MIP) rapidly lose colony forming ability and stop replating after about 3 weeks (Figure 5A). *Alox5*-/- cells transduced with MIP alone behave similar to their wildtype counterparts and display limited self-renewal. Interestingly, although RE9a-infected *Alox5*-/- cells initially replate longer than MIP control cells, after approximately 5 weeks in culture they begin to produce far fewer colonies than RE9a-infected wildtype cells and eventually lose replating capacity altogether (Figure 5A). These *Alox5*-/- cells also form smaller colonies as compared to wildtype (Figure 5B) and stain more positively for the myeloid differentiation marker CD11b when examined by flow cytometry (Figure 5C; Wildtype: 26–36%; *Alox5*-/-: 56–64%). These data indicate that lack of *Alox5* impairs the ability of RE9a to increase murine hematopoietic cell self-renewal, perhaps in part by altering cellular differentiation.

To determine whether the self-renewal defects observed in *Alox5*-/- cells were specific to RE9a or were more broadly applicable, we performed similar replating assays using the oncogenic fusion proteins MLL-AF9, resulting from t(9;11) and most frequently occurring in acute monoblastic leukemia (AML M5) [43], and PML-RARα, resulting from t(15;17) and observed in acute promyelocytic leukemia (APL/AML M3) [44]. Notably, decreases in colony numbers as compared to wildtype were observed after transduction of *Alox5*-/- cells with both MLL-AF9 and PML-RARα (Figure 5D–E), demonstrating that loss of *Alox5* impairs the increased self-renewal capability induced by multiple fusion oncoproteins.

It was previously reported that although *Alox5* is required for induction of CML by BCR-ABL, lack of *Alox5* in hematopoietic stem cells in the absence of BCR-ABL did not result in any significant hematopoietic defects [29]. Having observed that lack of *Alox5* also impaired increased self-renewal induced by RE9a, MLL-AF9 and PML-RARα, we next examined whether exogenous expression of *Alox5* itself had any effect. To test this, we transduced wildtype bone marrow cells with retrovirus encoding murine *Alox5* and performed serial replating assays. As shown in Supporting Figure S3, exogenous *Alox5* expression conferred no replating advantage relative to MIP-transduced control cells, demonstrating that while ALOX5 aids in increased self-renewal by multiple oncogenes, it is incapable of inducing this increase on its own.

### RE9a-expressing *Alox5*-/- cells are capable of leukemia induction

**Figure 6:**
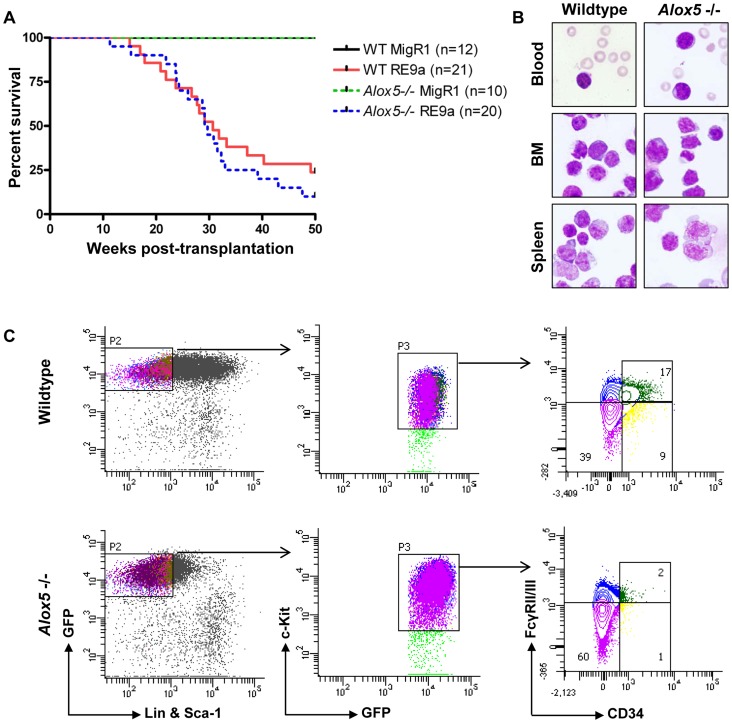
Loss of *Alox5* does not block RE9a leukemia induction *in vivo*. (A) Survival of mice receiving wildtype or *Alox5*-/- fetal liver cells transduced by control (MigR1) or RE9a retrovirus. Number of mice in each cohort shown at right. WT median survival: 30.71 weeks; *Alox5*-/- median survival: 29.43 weeks; *p* = 0.39. (B) Presence of hematopoietic blast cells in tissues of mice transplanted with RE9a-transduced wildtype or *Alox5*-/- cells. Peripheral blood smears and cytocentrifugation of bone marrow and spleen cells were stained with Wright-Giemsa solutions. (C) Immunophenotype of myeloid progenitor cells in wildtype and *Alox5*-/- leukemias. Distribution of EGFP^+^Lin^−^Sca-1^−^c-Kit^+^ leukemic cells harvested from spleen shown based on expression of CD34 and Fcγ receptors II/III (FcγRII/III). At least 4 mice analyzed per genotype, with representative distributions shown.

*Alox5*-/- hematopoietic cells transduced with multiple fusion oncogenes showed clear defects in self-renewal *in vitro* (Figure 5). It is important to determine whether these defects are also present *in vivo*. To investigate this possibility, we harvested wildtype and *Alox5*-/- fetal liver cells, retrovirally transduced them with virus encoding either RE9a-IRES-GFP or GFP alone (MigR1) and transplanted them into lethally irradiated recipient mice. As expected, neither wildtype nor *Alox5*-/- cells transduced with MigR1 induced leukemia (Figure 6A). In contrast, both wildtype and *Alox5*-/- cells expressing RE9a induced leukemia in recipient mice, with a median latency of approximately 30 weeks (Figure 6A). Both wildtype and *Alox5*-/- cells were also able to induce secondary and tertiary leukemias in further rounds of transplantations to new recipient mice (data not shown). Additionally, leukemic mice of both genotypes displayed similar blast cells in the peripheral blood, bone marrow and spleen (Figure 6B).

We have previously observed that RE9a-leukemic mice display a phenotype in their myeloid progenitor populations, in which the normal distribution of common myeloid progenitors (CMPs), granulocyte/monocyte progenitors (GMPs) and megakaryocyte/erythroid progenitors (MEPs) is lost, and a single population arises that is similar in immunophenotype to MEPs, but has increased expression of Fcγ receptors II/III (FcγRII/III) by flow cytometry [10]. Similar leukemia cells were also reported in an Inv(16) AML mouse model and termed abnormal myeloid progenitors [45]. To determine whether *Alox5*-/- leukemic cells also display this aberrant progenitor profile, we first examined untreated, non-leukemic bone marrow cells from wildtype and *Alox5*-/- mice and observed normal distributions of the 3 progenitor populations (Figure S4). We next checked the progenitor populations from leukemic mice. Although there were variations among both wildtype and *Alox5*-/- leukemic mice in terms of mean fluorescence intensity of GFP expression and percent of cells that were Sca-1 positive, we found that leukemic mice transplanted with either genotype of RE9a-infected cells displayed a similar abnormal myeloid progenitor immunophenotype (Figure 6C) as compared to controls (Figure S4). Some *Alox5-/-* leukemic mice displayed smaller GMP compartments than their wildtype counterparts (Figure 6C), but as the differences varied from mouse to mouse and had no effect on leukemia development (Figure 6A), we do not interpret this as being an important functional difference.

These results demonstrate that although loss of *Alox5* results in self-renewal defects *in vitro*, other factors exist *in vivo* that allow RE9a to overcome these defects and promote leukemia development.

## Discussion

In order to design better and more specific treatments for AML, we need a more thorough understanding of the underlying molecular mechanisms of cellular transformation that lead to disease. The 8;21 translocation that causes expression of the RUNX1-ETO DNA-binding fusion proteins is highly associated with AML M2, but its mechanisms of gene dysregulation are not completely understood. This is especially true for upregulated RE target genes. Here, we demonstrate that *Alox5* is an upregulated t(8;21) target gene and establish for the first time that KLF6 cooperates in transcriptional dysregulation with leukemia fusion proteins during target gene upregulation. Although the *Alox5* promoter is activated only weakly by wildtype RUNX1, both full-length RUNX1-ETO and its splicing isoform RUNX1-ETO9a strongly induce promoter activity, demonstrating that *Alox5* is upregulated by multiple t(8;21) fusion proteins (Figure S5).

ALOX5 is a promising molecular target for the treatment of CML, as it has been demonstrated that a small molecule inhibitor of ALOX5 significantly delays leukemia onset in mice [29]. We show here that lack of ALOX5 also leads to *in vitro* defects in hematopoietic cells transduced by RE9a, MLL-AF9 and PML-RARα, all of which are oncogenes involved in AML development. However, these results did not translate *in vivo*, as *Alox5*-/- cells infected with RE9a are still able to induce leukemia in mice (Figure 6A), and a similar result was obtained in a pilot experiment using an MLL-AF9 model of AML (data not shown). Additionally, no significant differences were observed in the differentiation states of wildtype and *Alox5*-/- leukemia cells, either in analysis of their progenitor populations or expression of lineage markers Gr1, CD11b, CD4 and B220 or progenitor markers c-Kit and Sca-1 (Figure 6C and data not shown). It is not entirely clear why the importance of ALOX5 demonstrated *in vitro* is not observed *in vivo*, although this difference in phenotype between the two systems has been observed previously. One such example is RUNX1-ETO itself, which induces a significant increase in hematopoietic self-renewal yet requires cooperating mutations to induce leukemia *in vivo* [46]. Additionally, lack of *Stat5* expression blocks the replating potential of the AML-inducing oncogene MOZ-TIF2 but only delays the onset of AML *in vivo* [47]. We observe a similar discrepancy for the requirement of *Alox5* between the *in vitro* and *in vivo* systems, for which multiple mechanisms may exist. One possibility arises from the fact that there are a limited number of factors present in the *in vitro* culture conditions. Additional factors or different concentrations of factors present *in vivo* may allow for *Alox5*-/- cell survival and transformation. In a CML model, BCR-ABL-expressing *Alox5*-/- leukemia stem cells (LSCs) display an increased apoptotic rate as compared to wildtype LSCs, indicating the potential importance of the stem cell population for *Alox5* and leukemia development [48]. Given this importance, it is possible that interactions between RE9a-infected stem cells and the niche *in vivo* allow them to survive and self-renew, whereas this interaction is absent *in vitro*. Another possible cause is cell-specific differences, as total bone marrow cells were used *in vitro* and fetal liver cells were used for the transplantation experiments. Recent studies indicate that the fetal liver and adult bone marrow contain differing ratios of hematopoietic stem cells (HSCs) with unequal differentiation potential, with the fetal liver specifically enriched for HSCs with long-term myeloid differentiation potential [49]. It is possible that different subtypes of HSCs respond differently to the introduction of RE9a, leading to the observed results. In addition, it is also possible that ALOX5 is required more by RE than RE9a in leukemia development, especially given that RE more strongly upregulates *KLF6* expression. Other plausible possibilities also exist. Therefore, the available evidence suggests that ALOX5 is not a suitable molecular target for the treatment of t(8;21) AML alone. Based on our replating data, it appears that ALOX5 does play a role in RE-induced increases in self-renewal. However, as this does not translate to a delay in leukemia onset *in vivo*, targeting ALOX5 alone is likely insufficient to generate therapeutic benefit, although its inhibition in combination with other treatments may show efficacy and this remains to be examined.

It is well established that RE represses target gene expression via the interaction of the ETO domain with N-CoR and SMRT and their associated histone deacetylases [50]. The mechanism by which RE upregulates gene expression is less well understood, although it was recently reported that the interaction between RE and p300 accounts for increased expression of at least some RE target genes and leukemogenicity [19]. Our current study has identified RE and RE9a as a novel positive transcriptional regulators of *Alox5*. Interestingly, the data indicate that regulation of *Alox5* by RE9a is indirect, as no peak of RE9a binding was observed in the *Alox5* promoter region when performing ChIP-chip on RE9a-leukemia cells [18], and no RUNX1 binding motifs are present in the RE9a-responsive region of the *Alox5* promoter (Figure 2). We further demonstrate that KLF6 is a critical factor for induction of the *Alox5* promoter in cooperation with RE9a. To the best of our knowledge, this is the first report demonstrating both KLF6 regulation of *Alox5* expression and the involvement of KLF6 in gene upregulation by t(8;21) fusion proteins. It will be important to examine whether this effect is specific to *Alox5* or if KLF6 more broadly participates in t(8;21)-mediated transcriptional alterations. If future work determines that KLF6 does in fact participate in the regulation of disease-related RE and RE9a target genes, KLF6 itself may become an interesting target for future study in the treatment of AML.

The involvement of KLF6 here is especially interesting in light of the fact that *KLF6* expression, like *ALOX5*, is also significantly upregulated in human AML M2 t(8;21)+ patient samples (Figure S1 and [34]). Therefore, KLF6 should be widely available in these leukemia cells to participate with t(8;21) fusion proteins in transcriptional regulation. We further demonstrate that increased *KLF6* expression is induced by introduction of both RE and RE9a into the non-t(8;21) AML M2 cell line HL60 (Figure 4). Interestingly, according to published ChIP-seq data, RUNX1-ETO binds multiple sites within and nearby the *KLF6* gene, indicating it may be a direct RUNX1-ETO target gene and this should be more closely studied in the future [16], [17].

Finally, we demonstrate for the first time that KLF6 interacts with RUNX1 and the Runt domain-containing t(8;21) fusion proteins. It is intriguing to note the potential implications of this interaction since, as with *Runx1*, knockout of *Klf6* in mice results in embryonic lethality with severe defects in differentiation across all hematopoietic lineages [33]. It will be interesting to examine a potential role of RUNX1 and KLF6 cooperation in early hematopoietic development. For instance, does RUNX1, like RUNX1-ETO9a, also co-regulate gene expression with KLF6 and if so, what are the functions of these genes in early hematopoiesis? Do KLF6 and RUNX1 function partially redundantly in hematopoietic development? Furthermore, KLF6 is only 1 of at least 9 KLF family members with important roles in blood cell function and disease development. KLFs 4, 5 and 10 are important in T-cell activation and trafficking and KLF4 may function as a tumor suppressor in adult T-cell leukemia, KLF2 promotes memory B-cell differentiation and KLF3-deficient mice display a myeloproliferative disorder [38]. It will be interesting then to determine whether RUNX1 or t(8;21) fusion proteins work with any of these other KLF family members in gene expression and normal or disease-related blood cell development.

## Materials and Methods

### Ethics statement

C57BL/6J wildtype and *Alox5-/-* mice used in this study were housed in a pathogen-free facility. All procedures were performed in strict accordance with the recommendations of the Institutional Animal Care and Use Committee of the University of California, San Diego, CA, and every effort was made to minimize suffering.

### Human and mouse gene expression

Human AML patient data [34] was analyzed using GraphPad Prism4 (GraphPad Software). For human cell line *KLF6* expression, total RNA was harvested from 10^6^ untreated HL60, Kasumi-1 and SKNO cells using the RNeasy Mini Kit (Qiagen). Retrovirally transduced HL60 cells were infected twice with virus produced by co-transfection of packaging vector and MSCV-IRES-Puro^r^ control or containing RUNX1-ETO in 293T cells. Infected HL60 cells were selected 2 days in 2 µg/ml puromycin to enrich for infected cells. 1 µg of RNA was used to generate cDNA using oligo (dT) and random primers (qScript cDNA SuperMix, Quanta Biosciences), and subject to qPCR on an iCycler (BioRad) using KAPA SYBR FAST Universal 2X qPCR Master Mix (KAPA Biosystems). *KLF6* primer sequences: forward: TTCTCGGCGCTGCCGTCTCT, reverse: TCGCCAATGGGGTCGGAGGTA. For mouse *Alox5* expression, lin^−^c-Kit^+^ hematopoietic cells were enriched from wildtype or leukemic mice using the Lineage Cell Depletion Kit and CD117 MicroBeads (Miltenyi Biotec) RNA extraction, cDNA synthesis and qPCR performed as above. *Alox5* primer sequences: forward: CTCTTCCAAGCTCGAAGTGC, reverse: TGATGCTACCGAGTGACGAG.

### Luciferase reporter assay

The indicated *Alox5* promoter regions were cloned into pGL2 vector (Promega) with the six consensus RUNX1 binding sites in the vector backbone mutated from TGTGGT to TGTtag (pGLX2) to prevent binding of RE9a to the vector [15]; 10^6^ K562 cells were nucleofected (Lonza) with 5 µg promoter-firefly luciferase DNA, 100 ng *Renilla* control luciferase DNA, and 2–3 µg p3xFlag-CMV-7.1 vector (Sigma-Aldrich) alone or containing RUNX1-ETO, RUNX1-ETO9a or KLF6 cDNA and analyzed 24 hr-post nucleofection using the Dual-Luciferase Reporter Assay System (Promega) on a Monolight 3010 (BD Biosciences). Unless otherwise stated, luciferase data show averages with standard deviations of 3 independent experiments, each performed in duplicate. For knock-down studies, 10^6^ K562 cells were first transfected with 6 µg pSUPER.retro.puro (Oligoengine) containing shRNA, selected 2 days in 2 µg/ml puromycin, and then transfected and analyzed as above. Hairpin sense-strand sequences: shKLF6#1: CGGCTGCAGGAAAGTTTAC; shKLF6#2: GGAGAAAAGCCTTACAGAT. Significance was determined by Student t-test.

### Immunoprecipitation and antibodies

K562 cells were nucleofected with 5 µg each p3xFlag-CMV-7.1-KLF6 and pcDNA6-HA-RUNX1, RUNX1-ETO or RUNX1-ETO9a. Pre-cleared lysates were incubated with rotation overnight at 4°C with 1 µg control or KLF6 antibody (Santa Cruz Biotechnology) and washed 5 times with lysis buffer [51] prior to SDS-PAGE. Antibody suppliers: α-tubulin (Covance), HA (Roche), SP1 (Santa Cruz), ALOX5 (Abcam).

### Retroviral transduction and replating assay

*Alox5*-/- mice[52] were purchased from Jackson Laboratory. Retroviral transduction and replating assays were performed as previously described [20]. Briefly, total bone marrow cells from wildtype or *Alox5*-/- mice were transduced with retrovirus MSCV-IRES-Puro^r^ (MIP) [53] vector control or MIP containing HA-RUNX1-ETO9a, murine HA-Alox5, MLL-AF9 or PML-RARα cDNAs, as indicated. Infected cells were selected 1 week in 1 µg/ml puromycin in M3434 (STEMCELL Technologies). Ten thousand cells from each transduction were replated in duplicate every 7 days after colony and cell counting.

### Fetal liver cell isolation, transduction, transplantation and flow cytometry

These assays were performed as previously described [10]. Briefly, fetal liver cells were harvested from day E13.5–16.5 wildtype or *Alox5*-/- mouse embryos and transduced twice with MigR1 or MIG-RUNX1-ETO9a retrovirus. Lethally irradiated (900 rad) wildtype recipient mice were intravenously transplanted with the transduced fetal liver cells. Gr-1, CD11b, Sca-1, c-Kit, CD34 and FcγRII/III fluorescently conjugated antibodies were purchased from eBioscience. Staining and analyses were performed as previously described [10]. Kaplan-Meier survival curves and statistical analyses were performed using GraphPad Prism4.

### Chromatin immunoprecipitation

ChIP assay was performed as described previously [20]. Cell lines were generated by retroviral transduction of K562 cells with MIP-HA-RE9a or MIP-Flag-KLF6. Each ChIP reaction contained chromatin from 10^7^ cells and 5 µg antibody. Antibodies used were: HA (Santa Cruz) for RE9a, Flag (Sigma-Aldrich) for KLF6, and N-terminal RUNX1 [10] for endogenous RUNX1 in the absence of exogenous RE9a and KLF6. Following immunoprecipitation, enrichment of regions of the *ALOX5* promoter was measured by qPCR using the following primers (numbers indicate nucleotides relative to transcription start site): *ALOX5*-A (−522 to −234) forward: AGCCTCTGTGCTCCAGAATCCATC, reverse: CGTTCACTCGTTCTCTCCTGAATTG; *ALOX5*-B (−259 to −79) forward: CAATTCAGGAGAGAACGAGTGAACG, reverse: GCAGTACTTCTCTCCCACTCTTCACG; *ALOX5*-C (+149 to +596) forward: CACTGACGACTACATCTACCTCAGCCTC, reverse: ATCTTGAAGTGGAGGGGAAACCTTG, and enrichment was normalized to IgG control.

## Supporting Information

Figure S1 *ALOX5* and *KLF6* expression in human AML patients. (A) Normalized log_2_ expression of *ALOX5* in human blast and mononuclear cells from bone marrow aspirates of AML subtype M2 patients with or without t(8;21) and in normal patient CD34+ samples. Patient data is from Valk *et al* [34]. Each point represents an individual patient sample. (B) Normalized log_2_ expression of *KLF6* in human AML subtype M2 patients as described in (A). n.s. = not significant. Patient samples were 80–100% blast cells at the time of analyses.
(PDF)

Figure S2 RE9a and KLF6 bind the human *ALOX5* promoter. Following ChIP, exogenous HA-RE9a and Flag-KLF6 show enrichment compared to IgG control at three locations within the *ALOX5* promoter. Endogenous RUNX1 shows no enrichment. Locations of three PCR amplicons relative to transcription start site of *ALOX5*: *ALOX5*-A −522 to −234, *ALOX5*-B −259 to −79, *ALOX5*-C +149 to +596.
(PDF)

Figure S3 *Alox5* in cellular self-renewal. Exogenous ALOX5 is insufficient increase cellular self-renewal on its own. Wildtype bone marrow cells were transduced with control (MIP), HA-ALOX5 or HA-RE9a retrovirus and serially replated in methylcellulose. Data shown are averages and standard deviations of a representative dataset. Three independent assays were performed. Expression of ALOX5 and RE9a in bone marrow cells after selection is shown by western blot (right). Tubulin serves as a loading control.
(PDF)

Figure S4 Myeloid progenitor profiles in untreated wildtype and *Alox5*-/- mice. Distribution of Lin^−^Sca-1^−^c-Kit^+^ bone marrow cells harvested from untreated mice shown based on expression of CD34 and Fcγ receptors II/III (FcγRII/III). Three mice analyzed per genotype, with representative distributions shown. GMP = Granulocyte/Monocyte Progenitor; CMP = Common Myeloid Progenitor; MEP = Megakaryocyte/Erythroid Progenitor.
(PDF)

Figure S5 Both RUNX1-ETO and RE9a strongly activate the *Alox5* promoter. Inducible regulation of *Alox5* promoter-luciferase by RUNX1 (A), RUNX1-ETO or RE9a (B). −507 to +146 reporter was co-transfected with control (Ctrl), RUNX1, RUNX1-ETO (RE) or RE9a and expression was normalized to *Renilla* luciferase, with Ctrl transfection normalized to 1. n.s. = not significant.
(PDF)
