# Supplementary material for: Cooperation between RUNX1-ETO9a and Novel Transcriptional Partner KLF6 in Upregulation of Alox5 in Acute Myeloid Leukemia
Source: PLoS Genet. 2013 Oct 10;9(10):e1003765. doi: 10.1371/journal.pgen.1003765 (PMC3794898; doi:10.1371/journal.pgen.1003765)
Supplement: Figure S1 — ALOX5 and KLF6 expression in human AML patients. (A) Normalized log2 expression of ALOX5 in human blast and mononuclear cells from bone marrow aspirates of AML subtype M2 patients with or without t(8;21) and in normal patient CD34+ samples. Patient data is from Valk et al [34]. Each point represents an individual patient sample. (B) Normalized log2 expression of KLF6 in human AML subtype M2 patients as described in (A). n.s. = not significant. Patient samples were 80–100% blast cells at the time of analyses. (PDF) [file pgen.1003765.s001.pdf]

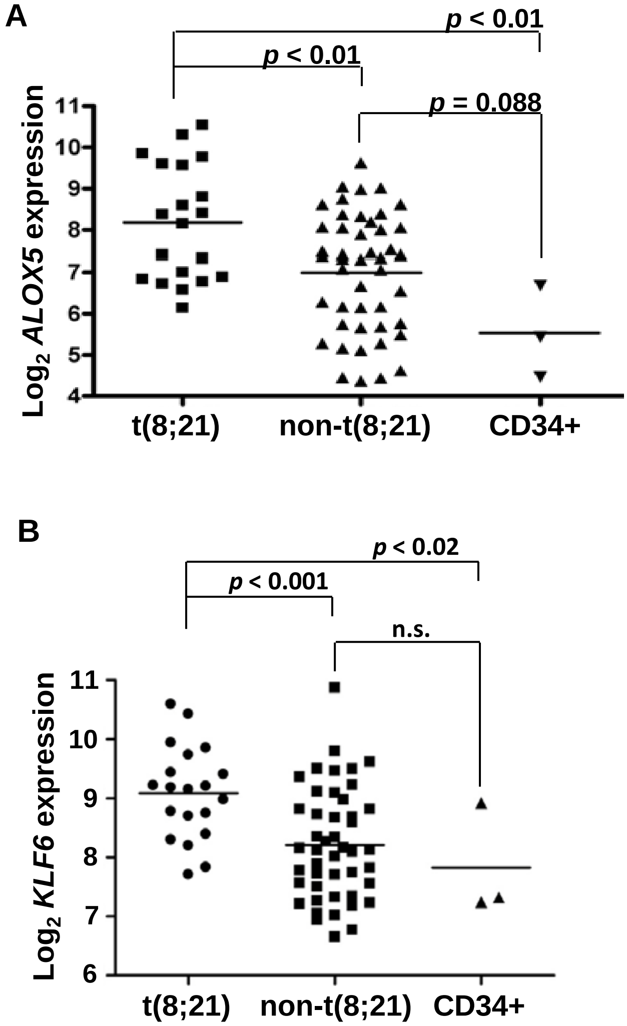

Supporting Figure S1. *ALOX5* and *KLF6* expression in human AML patients.
(A) Normalized $\log_2$ expression of *ALOX5* in human blast and mononuclear cells from bone marrow aspirates of AML subtype M2 patients with or without t(8;21) and in normal patient CD34+ samples. Patient data is from Valk *et al* [34]. Each point represents an individual patient sample. (B) Normalized $\log_2$ expression of *KLF6* in human AML subtype M2 patients as described in (A). n.s. = not significant. Patient samples were 80-100% blast cells at the time of analyses.
