# Supplementary material for: Cooperation between RUNX1-ETO9a and Novel Transcriptional Partner KLF6 in Upregulation of Alox5 in Acute Myeloid Leukemia
Source: PLoS Genet. 2013 Oct 10;9(10):e1003765. doi: 10.1371/journal.pgen.1003765 (PMC3794898; doi:10.1371/journal.pgen.1003765)
Supplement: Figure S2 — RE9a and KLF6 bind the human ALOX5 promoter. Following ChIP, exogenous HA-RE9a and Flag-KLF6 show enrichment compared to IgG control at three locations within the ALOX5 promoter. Endogenous RUNX1 shows no enrichment. Locations of three PCR amplicons relative to transcription start site of ALOX5: ALOX5-A −522 to −234, ALOX5-B −259 to −79, ALOX5-C +149 to +596. (PDF) [file pgen.1003765.s002.pdf]

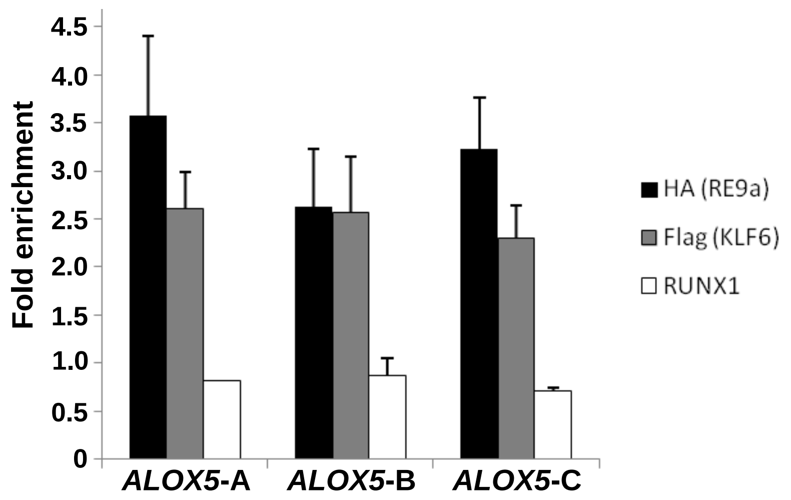

Supporting Figure S2. RE9a and KLF6 bind the human *ALOX5* promoter

Following ChIP, exogenous HA-RE9a and Flag-KLF6 show enrichment compared to IgG control at three locations within the *ALOX5* promoter. Endogenous RUNX1 shows no enrichment. Locations of three PCR amplicons relative to transcription start site of *ALOX5*: *ALOX5*-A -522 to -234, *ALOX5*-B -259 to -79, *ALOX5*-C +149 to +596.
