# Supplementary material for: Cooperation between RUNX1-ETO9a and Novel Transcriptional Partner KLF6 in Upregulation of Alox5 in Acute Myeloid Leukemia
Source: PLoS Genet. 2013 Oct 10;9(10):e1003765. doi: 10.1371/journal.pgen.1003765 (PMC3794898; doi:10.1371/journal.pgen.1003765)
Supplement: Figure S3 — Alox5 in cellular self-renewal. Exogenous ALOX5 is insufficient increase cellular self-renewal on its own. Wildtype bone marrow cells were transduced with control (MIP), HA-ALOX5 or HA-RE9a retrovirus and serially replated in methylcellulose. Data shown are averages and standard deviations of a representative dataset. Three independent assays were performed. Expression of ALOX5 and RE9a in bone marrow cells after selection is shown by western blot (right). Tubulin serves as a loading control. (PDF) [file pgen.1003765.s003.pdf]

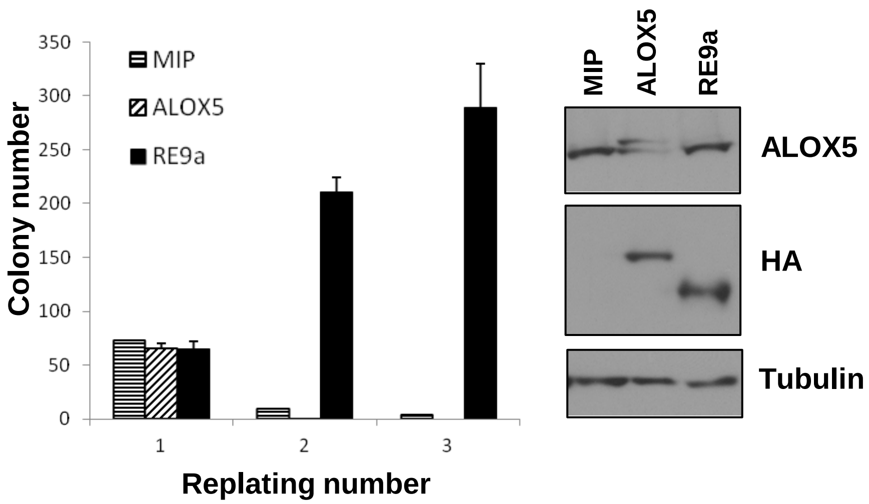

Supporting Figure S3. ***Alox5*** **in cellular self-renewal.**
Exogenous ALOX5 is insufficient increase cellular self-renewal on its own. Wildtype bone marrow cells were transduced with control (MIP), HA-ALOX5 or HA-RE9a retrovirus and serially replated in methylcellulose. Data shown are averages and standard deviations of a representative dataset. Three independent assays were performed. Expression of ALOX5 and RE9a in bone marrow cells after selection is shown by western blot (right). Tubulin serves as a loading control.
