# Supplementary material for: Cooperation between RUNX1-ETO9a and Novel Transcriptional Partner KLF6 in Upregulation of Alox5 in Acute Myeloid Leukemia
Source: PLoS Genet. 2013 Oct 10;9(10):e1003765. doi: 10.1371/journal.pgen.1003765 (PMC3794898; doi:10.1371/journal.pgen.1003765)
Supplement: Figure S4 — Myeloid progenitor profiles in untreated wildtype and Alox5-/- mice. Distribution of Lin−Sca-1−c-Kit+ bone marrow cells harvested from untreated mice shown based on expression of CD34 and Fcγ receptors II/III (FcγRII/III). Three mice analyzed per genotype, with representative distributions shown. GMP = Granulocyte/Monocyte Progenitor; CMP = Common Myeloid Progenitor; MEP = Megakaryocyte/Erythroid Progenitor. (PDF) [file pgen.1003765.s004.pdf]

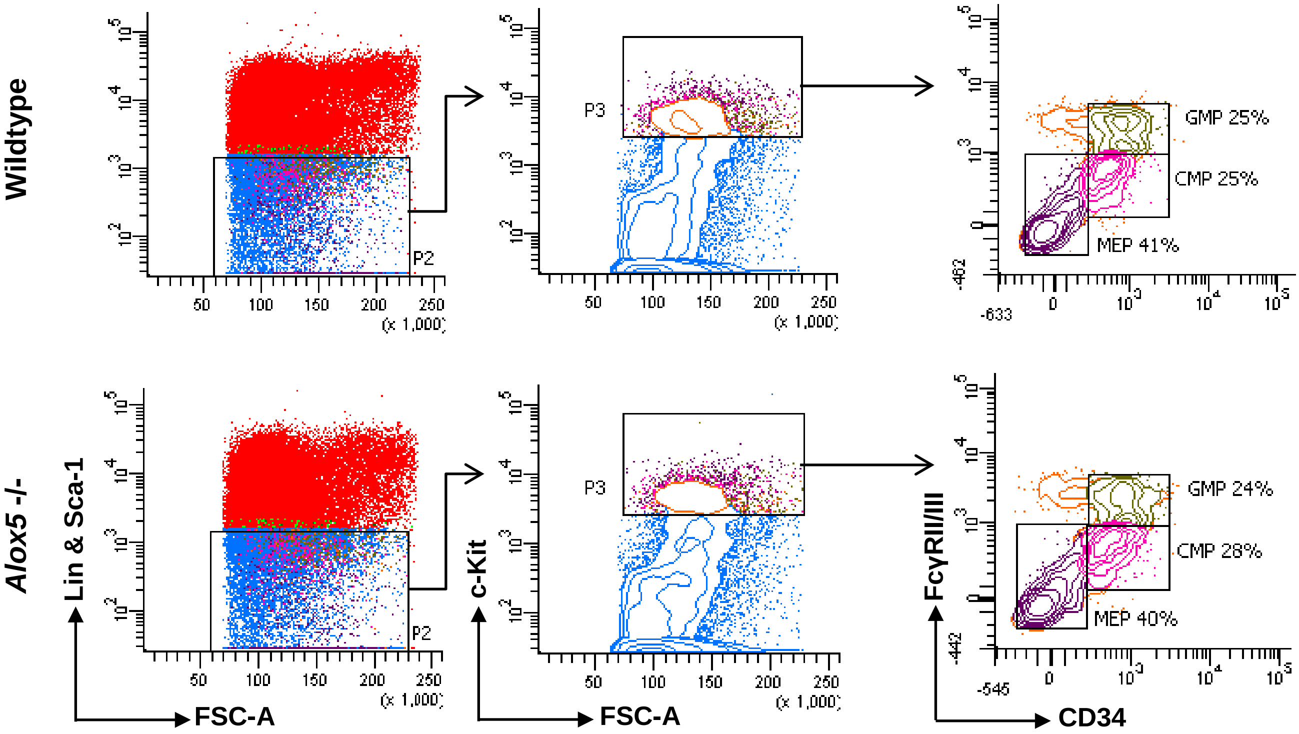

Supporting Figure S4. Myeloid progenitor profiles in untreated wildtype and *Alox5*-/- mice. Distribution of Lin⁻Sca-1⁻c-Kit⁺ bone marrow cells harvested from untreated mice shown based on expression of CD34 and Fcγ receptors II/III (FcγRII/III). Three mice analyzed per genotype, with representative distributions shown. GMP = Granulocyte/Monocyte Progenitor; CMP = Common Myeloid Progenitor; MEP = Megakaryocyte/Erythroid Progenitor.
