# Supplementary material for: Cooperation between RUNX1-ETO9a and Novel Transcriptional Partner KLF6 in Upregulation of Alox5 in Acute Myeloid Leukemia
Source: PLoS Genet. 2013 Oct 10;9(10):e1003765. doi: 10.1371/journal.pgen.1003765 (PMC3794898; doi:10.1371/journal.pgen.1003765)
Supplement: Figure S5 — Both RUNX1-ETO and RE9a strongly activate the Alox5 promoter. Inducible regulation of Alox5 promoter-luciferase by RUNX1 (A), RUNX1-ETO or RE9a (B). −507 to +146 reporter was co-transfected with control (Ctrl), RUNX1, RUNX1-ETO (RE) or RE9a and expression was normalized to Renilla luciferase, with Ctrl transfection normalized to 1. n.s. = not significant. (PDF) [file pgen.1003765.s005.pdf]

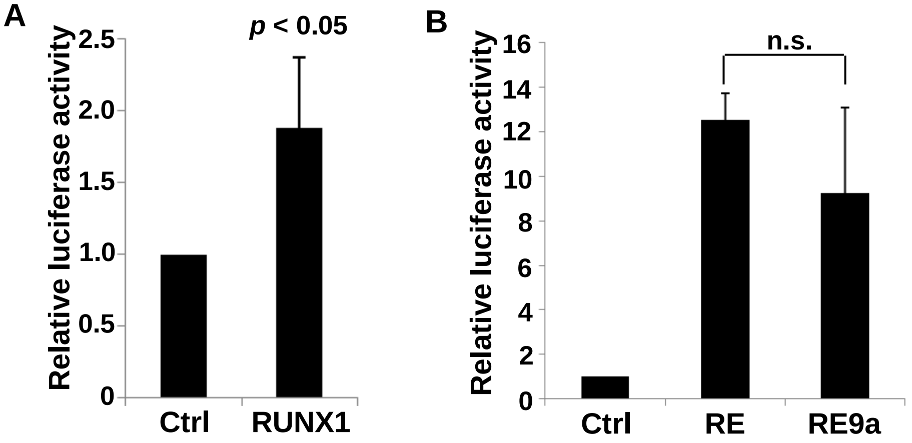

Supporting Figure S5. Both RUNX1-ETO and RE9a strongly activate the *Alox5* promoter
Inducible regulation of *Alox5* promoter-luciferase by RUNX1 (A), RUNX1-ETO or RE9a (B). -507 to +146 reporter was co-transfected with control (Ctrl), RUNX1, RUNX1-ETO (RE) or RE9a and expression was normalized to *Renilla* luciferase, with Ctrl transfection normalized to 1. n.s. = not significant.
